# Ranibizumab Modifies the Expression of Metalloproteinases and Their Tissue Inhibitors in Peripheral Blood Mononuclear Cells in Patients with Exudative Age-Related Macular Degeneration

**DOI:** 10.3390/jcm13010295

**Published:** 2024-01-04

**Authors:** Barbara Strzalka-Mrozik, Olga Paprzycka, Oliwia Gruszka, Marcel Madej, Celina Kruszniewska-Rajs, Joanna Magdalena Gola, Artur Turek

**Affiliations:** 1 Department of Molecular Biology, Faculty of Pharmaceutical Sciences in Sosnowiec, Medical University of Silesia, 40-055 Katowice, Poland; s78207@365.sum.edu.pl (O.P.); oliwia.gruszka@sum.edu.pl (O.G.); mmarcel281297@gmail.com (M.M.); ckruszniewska@sum.edu.pl (C.K.-R.); jgola@sum.edu.pl (J.M.G.); 2 Silesia LabMed, Centre for Research and Implementation, Medical University of Silesia, 40-752 Katowice, Poland; 3 Department of Biopharmacy, Faculty of Pharmaceutical Sciences in Sosnowiec, Medical University of Silesia, 40-055 Katowice, Poland; artur.turek@sum.edu.pl

**Keywords:** age-related macular degeneration, anti-VEGF treatment, ranibizumab, metalloproteinases, genes, oligonucleotide microarray, RT-qPCR

## Abstract

Background: Age-related macular degeneration (AMD) is the leading cause of vision loss in people over 60 years of age. Despite research, the causes of AMD remain unclear. Matrix metalloproteinases (MMPs) and their tissue inhibitors (TIMPs) are known to be involved in AMD development, and anti-vascular endothelial growth factor therapy has revolutionized its treatment. This study aims to analyze the changes in gene expression in MMPs and TIMPS in patients with neovascular AMD before and after three doses of ranibizumab. Methods: The study involved 29 patients with neovascular AMD treated with ranibizumab. Peripheral blood mononuclear cells were collected before treatment and 24 h after the third dose of ranibizumab. We assessed MMP and TIMP gene expression profiles through oligonucleotide microarrays and validated selected differential genes using RT-qPCR. Results: A statistically significant change in the expression of six MMP- and TIMP-related genes was observed using oligonucleotide microarray. The mRNA levels of the two genes with the most significant fold changes, *MMP15* and *TIMP2*, were then quantified using RT-qPCR. The results confirmed a statistically significant increase in *MMP15* expression and a decrease in *TIMP2* levels, although this change was not statistically significant in the group before and after the third dose of ranibizumab. Conclusion: Ranibizumab affects the systemic expression of MMP and TIMP-related genes in patients with neovascular AMD. Results from our exploratory study suggest that *MMP15*, in particular, may play a role in the treatment response, but further research is necessary.

## 1. Introduction

Age-related macular degeneration (AMD) is the leading cause of vision loss in older adults, irrespective of gender. In 2020, statistics indicated that AMD affected just over 190 million people globally and the number of cases is expected to rise to around 288 million by 2040 [1,2,3]. In Poland, according to various sources, between 1.2 and 1.9 million people suffer from AMD, of which 130,000 to 140,000 have the more severe form known as wet AMD (wAMD). It is estimated that between 14,000 and 20,000 new cases of wAMD occur each year. A 2016 report by the Institute of Healthcare suggested that in Poland, the number of AMD patients in 2016 may have reached 2.7 million people, including 273,000 with wAMD. Furthermore, projections indicate that this number could increase to as many as 3.6 million by 2060 [4,5].

The disease leads to a gradual loss of central vision, which is attributed to pathological changes in the retinal layers and adjacent blood vessels [6]. While the precise mechanisms underlying AMD pathogenesis are not yet fully understood, there is accumulating evidence suggesting a pivotal role for extracellular matrix (ECM) dysregulation in its development and progression. Genetic and environmental factors that affect the synthesis of ECM regulatory elements are also under consideration [7,8].

Vascular endothelial growth factor (VEGF) is a potent cytokine that modulates angiogenesis. However, it is equally important to remodel the extracellular matrix to create conditions favorable to the expansion of newly formed vessels. Intriguingly, the dysregulation of matrix metalloproteinases (MMPs) and tissue inhibitors of metalloproteinases (TIMPs) may impact its expression. In turn, an increased VEGF level, triggered by hypoxia, can positively regulate MMP2 and MMP9 secretion and influence choroidal neovascularization (CNV) in AMD, leading to serious visual impairment [7,9,10,11]. Additionally, VEGF-A increases the expression of MMP15 and ADAMT, both of which contribute to abnormal angiogenesis [9].

ECM consists of stroma (matrix) and basement membranes that stretch between the matrix and the cells. It serves as a supportive framework for cells and provides an internal environment that facilitates the diffusion of nutrient molecules and metabolic byproducts. Furthermore, the ECM regulates signal conduction and offers protection against external undesirable factors [7,9].

A total of 23 MMPs, categorized both numerically and based on substrate decomposition, play pivotal roles in the continual digestion and rebuilding of the ECM. They are crucial in regulating various cellular processes, including angiogenesis (cellular proliferation, differentiation, migration, and adhesion), apoptosis, inflammatory reactions, and the activities of cell-to-cell adhesion molecules, integrin, growth factors, cytokines, and chemokines [12,13]. The production and secretion of these MMPs are genetically regulated [14,15].

Due to the diverse processes catalyzed by MMPs, their activity is negatively regulated by four endogenous tissue inhibitors of metalloproteinases, namely TIMP1, TIMP2, TIMP3, and TIMP4. An increase in MMP concentration triggers TIMP synthesis [12,13]. While TIMPs inhibit MMPs non-selectively, they are highly selective in inhibiting the ADAM/ADAMTS family [15].

Changes in the ECM, such as degradation or accumulation of its components, are linked to disruptions in MMP/TIMP complexes. Such disruptions in TIMP- and MMP-dependent ECM remodeling are associated with the loss of function in photoreceptors and retinal pigment epithelial cells, as well as chronic inflammation and neovascularization [13].

The anatomical and functional complex involved in exudative AMD includes photoreceptors, retinal pigment epithelium (RPE) cells, the extracellular matrix—specifically the 2–4 µm thick Bruch’s membrane (BrM)—and choriocapillaris. This complex is characterized by the presence of soft drusen (SD) and CNV in both early and late stages of exudative AMD [7,9].

During aging, there is an accumulation of ECM material, accompanied by changes in MMP and TIMP levels in the BrM. These alterations in enzyme expression have the potential to contribute to the development of AMD. Notably, early stage AMD is characterized by the presence of extracellular deposits, which have been observed in cases of oxidative damage to the retina, coinciding with a decrease in MMP2 levels [10,11]. Neovascular AMD has been linked to MMP2 and MMP9 in the Bruch membrane, with MMP9 promoting a proangiogenic environment.

Importantly, an increase in MMP14 levels serves a protective role by preventing oxidative damage to the retina through the regulation of MMP2 activity [10,11]. Furthermore, in response to cellular oxidative stress, the levels of MMP3 and MMP-1 also increase. The imbalance in MMP1-3/TIMP1 concentrations can lead to type 1 collagen degradation, potentially triggering early wet AMD [10,11].

It was also shown that changes in the levels of TIMP suggest that it is related to the pathogenesis of AMD. TIMP3 naturally occurs in the BrM, with the RPE being the primary site for *TIMP3* gene expression. Mutations in the *TIMP3* gene result in the production of aberrant protein, leading to Sorsby’s eye dystrophy, a hereditary form of early-onset AMD. Studies have demonstrated an age-associated increase in TIMP3 levels in ocular tissues in AMD patients. Intriguingly, immunohistochemical assays using anti-TIMP3 antibodies have detected its presence in extracellular deposits [16]. In AMD patients, a correlation between TIMP1, MMP9, and geographical atrophy has also been observed [6].

The inhibition of neovascularization formation is crucial to the treatment of exudative AMD. Intraocular injection of VEGF-inhibiting drugs has become the primary method of treating exudative AMD [17]. Ranibizumab, an anti-VEGF drug, is currently the first-line therapy for CNV/AMD, with its effectiveness validated by pharmacokinetic studies and clinical trials [18,19]. The mechanism of action of ranibizumab, which is an antibody targeting VEGF-A, involves the neutralization of VEGF-A within neovascular lesions [20]. At the molecular level, anti-VEGF therapy influences gene expression, including genes related to ECM [18]. Other registered drugs include pegaptanib, bevacizumab, aflibercept, conbercept, and brolucizumab [17].

Bimonthly or monthly intravitreal injections of aflibercept, after an initial three-month dose, have shown comparable safety and tolerability to monthly injections of ranibizumab [17,21,22].

Conbercept is composed of the human VEGFR-1 and VEGFR-2 binding domains along with the Fc portion of human immunoglobulin G-1 and exhibits potent anti-angiogenic activity [17,23,24]. Intravitreal injection of conbercept was found to be safe and effective in the prevention of lesion development and CNV.

Brolucizumab is a single-stranded humanized antibody that targets VEGF and binds to the VEGF-A subtype. In contrast, faricimab is a bispecific antibody capable of binding to and inactivating angiopoietin-2 (Ang-2) and VEGF-A. The activity of faricimab is similar to that of ranibizumab [17,25].

Abicipar pegol is an anti-VEGF molecule that uses the technology of an engineered ankyrin repeat protein (darpin) to reduce VEGF levels. It has a longer half-life in the eye and provides a more persistent effect [17,26].

At least 13 drugs with VEGF-related targets are still in clinical development. Abicipar pegol, Faricimab, TypeOPT-302, and Dorzolamide-timolol are in phase III clinical trials; RGX-314, GB-102 (Sunitinib), X-82 (Vorolanib), HMR59 (AAVCAG-sCD59), CM082 tablets, and RG7716 are in phase II clinical trials; IBI302, ADVM-022, and TAB014 are in phase I clinical trials [17]. Although intravitreal anti-VEGF therapy initially stabilizes vision, long-term outcomes are less promising. It is important to recognize that AMD is a vascular-related disease, and even though the treatment primarily targets the eyes, its effects can have systemic implications. Previous research suggested that this therapy may lead to serious cardiovascular complications [27,28]. There is also an indication that anti-VEGF agents may potentially reach the contralateral (opposite) eye through the systemic circulation [29,30]. 

Analyzing gene expression profiles enables the investigation of systemic biomarkers, offering insights into the treatment’s impact on the entire organism. Measuring metalloproteinases in the ECM can serve as a valuable general marker for AMD, given their documented expression levels in blood cells associated with eye diseases. An added advantage of measuring MMPs, TIMPs, and ADAMTs is the relatively non-invasive nature of the procedure [10,12]. Moreover, they can potentially serve as markers of treatment response [18]. Unfortunately, there is limited research on the levels of MMPs, TIMPs, and ADAMTs in the blood of AMD patients, as well as a scarcity of studies examining their expression levels before and after treatment with anti-VEGF antibodies. 

Therefore, the aim of this study was to evaluate differences in the expression patterns of genes encoding matrix metalloproteinases and their tissue inhibitors in patients with CNV/AMD before and after the administration of a loading dose of ranibizumab. This research has the potential to enhance patient care and advance our comprehension of the molecular mechanisms at play in AMD.

We focused specifically on *MMP15*, an activator of ADAMTS proteases 1, -2, -4, -5 and -13, which have been implicated in the development of eye diseases. *TIMP2* is a regulator of *MMP2* gelatinase which is responsible for the degradation of collagen, gelatin and elastin. Both *TIMP2* and the gelatinase it regulates have been detected in neovascular choroidal membranes [31,32,33].

## 2. Materials and Methods

### 2.1. Materials

The study utilized peripheral blood samples from Polish patients undergoing treatment with ranibizumab. Blood samples were obtained from the ulnar vein, with each sample consisting of 5 mL of blood. Sampling occurred at two different time points: 24 h prior to the initial ranibizumab dose and 24 h after the third ranibizumab injection. To minimize potential diurnal variations in gene expression profiles, blood collection took place in the morning, specifically between 8:00 a.m. and 10:00 a.m. Ethylenediaminetetraacetic acid (EDTA)-containing tubes were used to collect the blood samples, preventing clotting.

Subsequently, the collected blood was divided into two equal parts and promptly diluted with an equivalent volume of phosphate-buffered saline (PBS). This dilution step aimed to reduce blood viscosity and facilitate the separation of peripheral blood mononuclear cells (PBMCs) using Ficoll–Conray density gradient centrifugation.

### 2.2. Test Group

The criteria for participation in the study and intravitreal ranibizumab injections were qualified ≥60 years old patients with decreased BCVA (20/25 to 20/200 Snellen equivalent) lasting no longer than 3 months and caused by previously untreated and active sub foveal CNV/AMD, confirmed by indirect mydriasis biomicroscopy (+78 D lens; Volk Optical, Mentor, OH, USA) and six diagonal high-density 6 mm scans at 30-degree intervals of optical coherence tomography (Stratus III OCT; Carl Zeiss, Dublin, CA, USA). Fundus fluorescein angiography without or with indocyanine green angiography (5 mL 10% fluorescein/25 mg indocyanine green administered intravenously; Fundus Camera FF 450 plus IR; Carl Zeiss) was performed in each patient to determine the type of CNV. Participants were carefully selected to exclude individuals with comorbidities such as hypertension, recent ophthalmic surgery, stroke, or myocardial infarction within the past 6 months.

Patients were excluded if they had retinal neovascularization due to other pathologies, such as polypoidal choroidal vasculopathy, retinal angiomatous proliferations, idiopathic CNV, or CNV resulting from high myopia, angioid streaks, inflammation or infection, choroid tumors, injuries, or retinal vein occlusion. Additional exclusion criteria included coexisting diabetic maculopathy and/or epiretinal membrane. 

The study group comprised 29 patients with diagnosed neovascular AMD, with a mean age of 76.8 years (Table 1).

All participants provided written consent for both the ranibizumab therapy and their participation in the study. 

The study was approved by the local Bioethics Committee of the Faculty of Medicine of the University of Warmia and Mazury in Olsztyn (resolution number 58/2019, dated 27 June 2019).

### 2.3. Intravitreal Injections

Intravitreal injections of 0.5 mg/0.05 mL ranibizumab were administered according to the PrONTO scheme, which consists of an initial series of three monthly injections, followed by additional reinjections based on CNV activity. All procedures were carried out by a single ophthalmologist, following aseptic techniques and using infiltration anesthesia for patient comfort. 

The combination of a standardized intravitreal injection procedure and a structured post-injection monitoring and follow-up protocol allowed for the effective evaluation of ranibizumab treatment in patients with AMD. These measures ensured patient safety, treatment efficacy, and the collection of comprehensive clinical data for analysis.

Treatments were conducted at the Department of Ophthalmology, University of Warmia and Mazury, Poland.

### 2.4. Preparation of Material and Molecular Analysis

The molecular studies began with the isolation of peripheral blood mononuclear cells using Ficoll–Conray density gradient centrifugation. This process lasted for 30 min at 1500 revolutions per minute (rpm) at room temperature, utilizing a solution with a specific gravity of 1.077 (Immunobiological Co., Gunma, Japan). This procedure was performed promptly after blood collection, and the isolated PBMCs were then stored at −80 °C for a duration of 24 h before RNA extraction took place.

Subsequently, total RNA was extracted from the PBMCs of patients with exudative AMD who were undergoing treatment with ranibizumab. This extracted RNA served as the template for transcriptome analysis, which was performed using the HGU-133A 2.0 oligonucleotide microarray method (Affymetrix, Santa Clara, CA, USA). The results were further validated using real-time RT-qPCR. The study also involved both qualitative and quantitative assessments of the transcriptomes.

### 2.5. Ribonucleic Acid Isolation and Qualitative and Quantitative Evaluation of the Extracts Obtained

Total RNA was extracted from the study samples using the Total RNA Prep Plus kit (A&A Biotechnology, Gdansk, Poland), according to the manufacturer’s guidelines. This extraction method is based on the Chomczynski and Sacchi method [34] and is known for its ability to effectively isolate RNA from PBMCs while maintaining RNA integrity. Subsequently, RNA purification was carried out using the RNeasy Mini Kit (Qiagen Inc., Hilden, Germany, Cat. No. 74106) in conjunction with DNase I (RNase-Free DNase Set, Qiagen Inc., Cat. No. 79254).

The quantity of the obtained RNA was assessed spectrophotometrically using a MaestroNano MN-913 nanospectrophotometer (MaestroGen Inc., Las Vegas, NV, USA). For qualitative evaluation, electrophoresis was performed on a 1% agarose gel stained with SimplySafe (EurX, Gdańsk, Poland). The electrophoretic pattern was then analyzed using a UV transilluminator as part of the Biotec-Fischer BaSys 1D gel documentation system (Biotech-Fischer, Perth, Australia).

### 2.6. Evaluation of Gene Expression Profile Using Oligonucleotide Microarray Technique

Gene expression profiling was conducted using the HG-U 133A 2.0 oligonucleotide microarray (Affymetrix, Santa Clara, CA, USA), according to the manufacturer’s recommendations. The methodology for transcriptome analysis using this oligonucleotide microarray has been previously outlined [10].

### 2.7. Reverse Transcription-Quantitative Polymerase Chain Reaction

The microarray results were validated using real-time RT-qPCR in all study participants. Each reaction was performed in triplicate. The quantitative analysis was conducted with SYBR Green I chemistry (SYBR Green Quantitect RT-PCR Kit, Qiagen, Valencia, CA, USA), in accordance with the manufacturer’s recommendations, on a DNA Engine Opticon™ System (MJ Research Inc., Watertown, MA, USA). The thermal profile for one-step RT-PCR was as follows: 45 °C for 10 min for reverse transcription, 95 °C for 2 min, 40 cycles at 95 °C for 5s, 60 °C for 10s, and 72 °C for 5s. Each run was completed using melting curve analysis to confirm the specificity of the amplification and the absence of the primer dimers. The specifications for the primers (Forward and Reverse) used in amplification at a final concentration of 500 nM are presented in Table 2. The *ACTB* gene served as an internal control for amplification, while non-matrix samples acted as negative controls. The mRNA copy number was determined using the absolute quantification method, and the results were calculated based on a standard curve, as previously described by Strzałka-Mrozik et al. [19]. Finally, the results obtained for mRNA copy numbers were recalculated per 1 μg of total RNA.

### 2.8. Statistical Analysis

Oligonucleotide microarray data were analyzed using the PL-Grid Infrastructure (http://www.plgrid.pl/), utilizing the Gene Spring 13.0 platform (Agilent Technologies UK Limited, South Queensferry, UK). Data normalization was performed using the Robust Multi-Array Average method. Differences in the expression levels of the studied transcripts between the two groups were assessed using an unpaired t-test with a cutoff of at least a 2.0-fold change and *p* < 0.05.

For the real-time RT-qPCR results, statistical analyses were conducted using Statistica v.10.0 (StatSoft, Tulsa, OK, USA). The normality distribution of the obtained data was initially assessed using the Shapiro–Wilk test, and the Dixon test was employed to detect outliers in continuous variables. The U Mann–Whitney non-parametric test was employed to evaluate statistical significance and differences between groups. The analysis used the generally accepted level of statistical significance in the study, *p* < 0.05. The values obtained through real-time RT-qPCR were expressed as medians with the 25th and 75th quartiles, as well as minimum and maximum values.

## 3. Results

### 3.1. Analysis of Results Obtained from Oligonucleotide Microarrays

Oligonucleotide microarray analysis, employed as a screening method, was conducted on 8 out of 29 blood samples from patients receiving treatment for wet AMD—3 samples were collected before drug administration and 5 samples were taken after three ranibizumab injections.

Each gene chip used in this study featured 22,277 probe sets. We focused on comparing the transcriptomes of PBMCs in patients before and 24 h after the third drug injection, specifically targeting differential IDs for mRNAs related to matrix metalloproteinases and their tissue inhibitors.

For this purpose, 110 mRNA identifiers indicated below were excluded from the Affymetrix Analysis Center NetAffx database (http://www.affymetrix.com/analytic/index.affx, access date: 15 March 2023). Significant differences in expression were observed in 16 of these mRNA IDs (*p* < 0.05). Furthermore, six mRNA IDs displayed a statistically significant change in expression level with a fold change (FC) ≥ 2.0. Details of these mRNA IDs are presented in Table 3.

Differences in gene expression profiles related to metalloproteinases and their tissue inhibitors were visualized using heat maps generated through GeneSpring XG. Changes in gene expression were assessed based on the color shifts in fluorescence signals. In the heat maps, blue represents the lowest fluorescence signals, while red corresponds to the highest.

**Figure 1:**
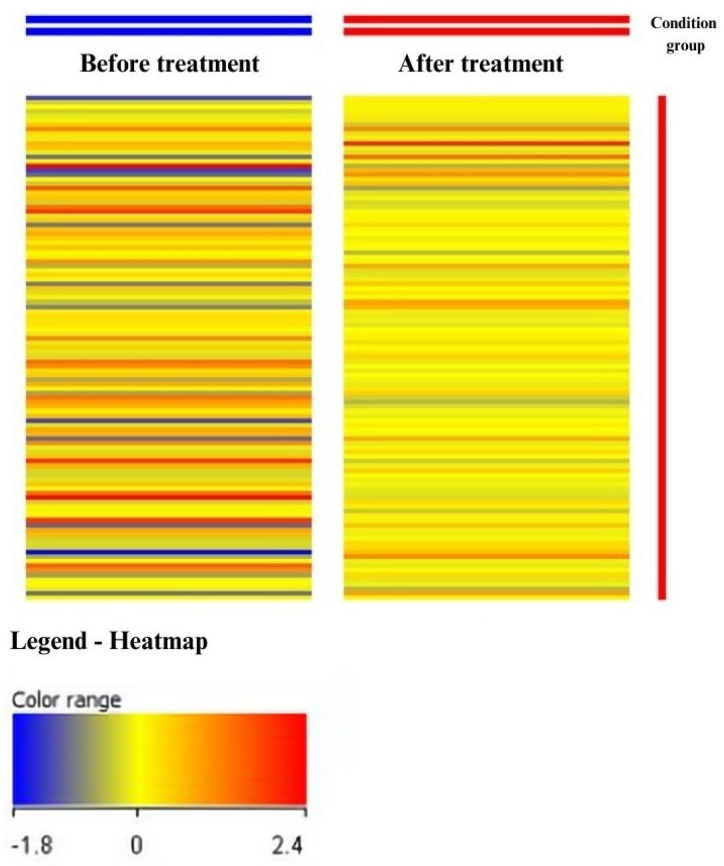
Part of the heat map of fluorescence signals for 110 ID mRNAs associated with MMPs and TIMPs in the study groups. The color variation between transcriptome groups indicates the presence of differences in gene expression profiles. Red color—higher values of fluorescence signals, high gene expression; blue color—lower values of fluorescence signals, low gene expression; before treatment—the group before the administration of ranibizumab; after treatment—the group after three injections of ranibizumab. Figure was generated using the software GeneSpring XG v.14.9, Agilent.

Based on the color change of the fluorescence signals, we evaluated the change in the expression of the studied genes. On these heat maps, blue signifies the lowest and red indicates the highest fluorescence signals (Figure 1). A different color pattern was observed in each study group (before and 24 h after the third injection of ranibizumab), indicating differences in gene expression profiles between them.

**Figure 2:**
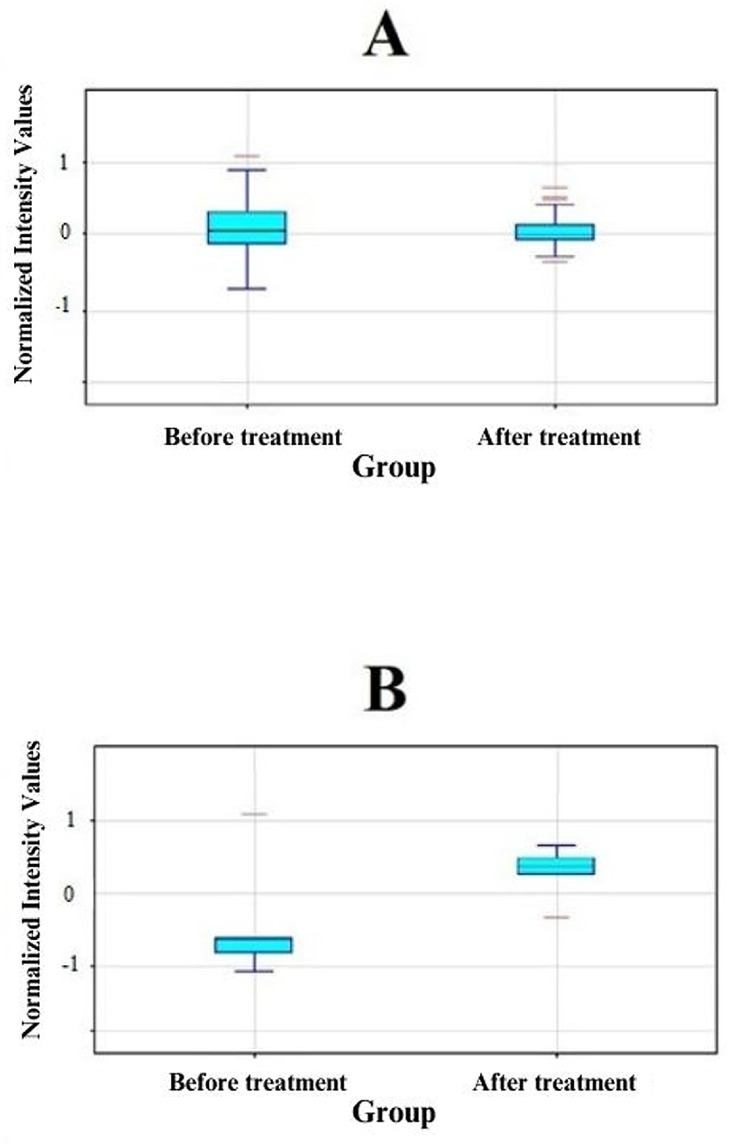
Graph of normalized fluorescence intensities for 110 ID mRNAs associated with MMPs and TIMPs (**A**) and for 6 ID mRNAs that showed a more than 2-fold statistically significant change in expression levels (**B**) in the study groups. Medians—black horizontal lines; quartile range value—height of the rectangle (light blue color) and minimum and maximum values; red color—signals deviating from the quartile range value by at least 1.5 times; before treatment—the group before the administration of ranibizumab; after treatment—the group after three injections of ranibizumab. Figure was generated through software GeneSpring XG v.14.9, Agilent.

Further validation of these transcriptomic variations within individual study groups is supported by the descriptive statistics of fluorescence signals for the 110 selected mRNA IDs related to metalloproteinases and their tissue inhibitors and the 6 mRNA IDs, with more than a 2-fold change in expression (Figure 2).

The differential transcripts associated with MMPs and TIMPs for the group before and the group 24 h after the third dose of the ranibizumab, along with the values of the FC parameter and the direction of the observed change, are shown in Table 4.

### 3.2. Analysis of Results Obtained by Real-Time RT-qPCR

Based on the results of the oligonucleotide microarray analysis, we chose to validate only two genes using an independent real-time RT-qPCR method: *TIMP2* and *MMP15*. *TIMP2* and *MMP15* were selected as genes that showed the highest fold change in fluorescence signal in the analysis. The choice was also confirmed by previous research [35,36,37,38].

Total RNA extracted from PBMCs of 29 patients, both before and 24 h after administration of the third dose of ranibizumab, was used to conduct the RT-qPCR analyses.

**Figure 3:**
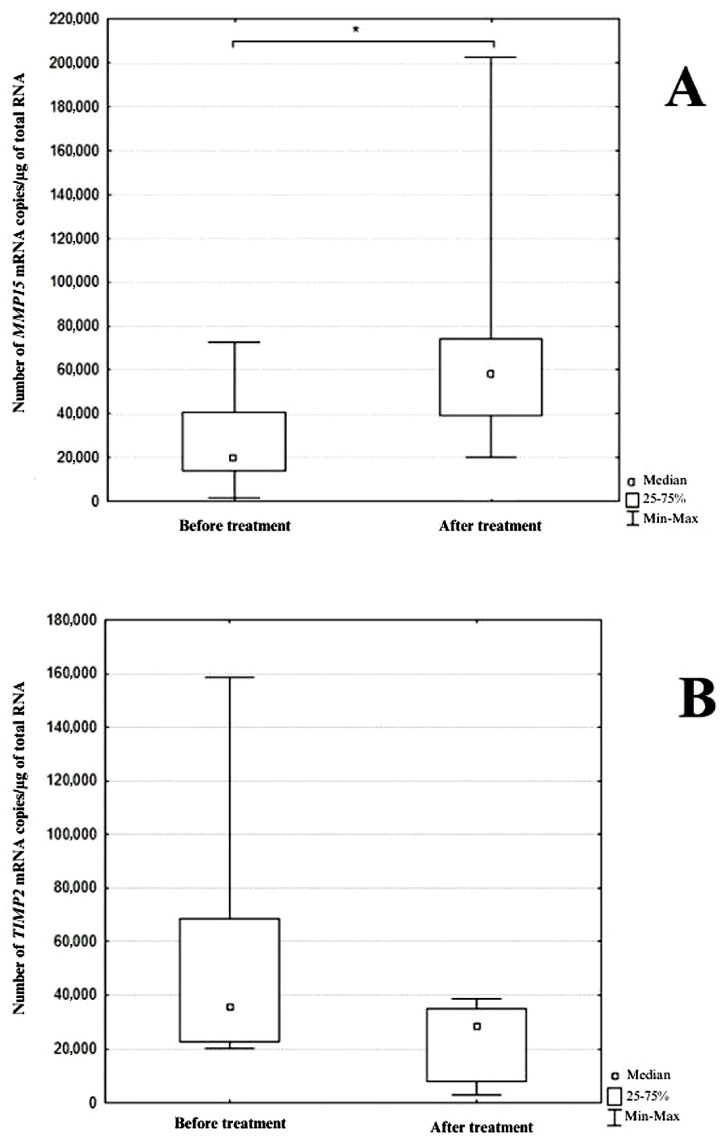
Comparison of the number of mRNA copies/µg of total RNA for: *MMP15* (**A**), *TIMP2* (**B**) in the study groups. Box and whisker plots present medians ± quartiles and extreme values of copy numbers per 1 μg of total RNA; U Mann–Whitney test; *—statistical significance (*p* < 0.05); before treatment—the group before the administration of the ranibizumab; after treatment—the group after three injections of ranibizumab. Figure was generated using the software Statistica v.10.0, StatSoft.

Transcriptional activity for both the *MMP15* and *TIMP2* genes was detected in all patient samples. Due to the non-normal distribution of the data, the results were reported as the median ± interquartile range (Me ± IQR). The number of mRNA copies/µg RNA before ranibizumab administration was provided for *MMP15*—19,841.88 ± 26,764.30 and for *TIMP2*—35,733.05 ± 45,847.62 mRNA copies/µg RNA, while 24h after the third dose of ranibizumab the mRNA copy number for the *MMP15* gene was 57,996.92 ± 34,554.84 and for the *TIMP2* gene, it was 28,528.63 ± 26,707.83 mRNA copies/µg RNA. A statistically significant difference in the expression level of the *MMP15* gene was observed between the group sampled before drug administration and the group sampled 24 h after the third dose of ranibizumab (Mann–Whitney U test; *p* = 0.002). In contrast, for the *TIMP2* gene, although a trend toward significant changes in expression levels was noted between the analyzed groups, the differences did not reach statistical significance (Mann–Whitney U test; *p* = 0.090). The results were graphically represented using a box plot that displayed the median, first and third quartiles, as well as the minimum and maximum values (Figure 3).

## 4. Discussion

The constantly increasing number of patients with diseases typical of old age encourages researchers to look for effective methods of prevention and treatment of these diseases. One example would be age-related macular degeneration, an eye disease characterized by progressive loss of vision central that is one of the main causes blindness in people over 50 years of age in developed countries [27].

The pathogenesis of AMD is not fully understood, but it is multifactorial and includes the interactions of genetic, metabolic, environmental, and functional factors [39,40]. Many factors have already been indicated that play a role in its development, but apart from age, there is no evidence to clearly determine the most important of them. Therefore, the disease is still being analyzed, especially in terms of molecular factors, the detection of which could facilitate its treatment and guarantee the effectiveness of preventive methods. It seems that analysis of changes in the expression of various genes, signaling pathways and factors that modulate them seem to be crucial and underlie this disease [7,9,41].

The results of our pilot study on a highly homogeneous group of patients in terms of age, race and gender, suggest a specific involvement of matrix metalloproteinases and their inhibitors in the pathogenesis of neovascular AMD. Our control group comprised AMD patients before ranibizumab administration, while the study group was composed of the same patients but after ranibizumab administration at specific time intervals. The primary focus was on their quantitative relationship to MMPs and TIMPs. The analysis indicates an increase in the expression of some MMPs and a decrease in the expression of one of the TIMPs. This imbalance leads to increased degradation of the extracellular matrix. While such an effect is undesirable in normal tissue, as it may contribute to disorders and even carcinogenesis, it may be beneficial for patients with neovascular AMD who have accumulated excessive extracellular material [9,10]. For these patients, a high MMP/TIMP ratio could potentially improve their health and quality of life.

Zhao et al. [20] studied the key genes associated with the AMD disease process. Comparative microarray analyses were performed to identify differentially expressed genes (DEGs) between individuals with AMD and healthy controls. The primary goal of this study was to identify the molecular pathways involved in the development of AMD and to propose new therapeutic approaches. The study material was obtained from three AMD patients (aged 66, 77 and 78) and three healthy individuals. The microarray results were validated through RT-qPCR. Eight genes were identified, including those that encode for fibroblast activation protein alpha (FAP), tenascin-C (TNC), glutathione peroxidase 3 (GPX3), gastrin-releasing peptide (GRP), metalloproteinase ADAM with thrombospondin type 1 motif 5 (ADAMTS5), TNF receptor-associated factor 6 (TRAF6), 7-dehydrocholesterol reductase (DHCR7), and farnesyl diphosphate farnesyl transferase 1 (FDFT1) [20,41]. The results of this study are partially consistent with our observations regarding the increase in *ADAMT* gene expression. It is important to note that differences may arise from the distinct approach of our study compared to the cited research [20]. While the referenced study compares results between patients with and without AMD, our study focuses on analyzing the changes in patients with neovascular AMD before and after three injections of ranibizumab. Additionally, variations in results may be attributed to differences in the size of the study groups.

In our study using oligonucleotide microarrays, we found that the levels of *MMP11*, *MMP14*, *MMP15*, *MMP24-A*, and *ADAMTS7* were significantly upregulated, while *TIMP2* was downregulated in the PBMCs of AMD patients after receiving three injections of ranibizumab. These differences could be attributed to various factors, including the research model employed and the number of samples analyzed.

Another study that aligns with this research approach was conducted by Yoshida et al. [42], who examined the gene expression profile in the human retina. Their study involved two age groups: individuals aged 13–14 and those aged 62–72. Comparative analyses revealed that retinal aging is linked to changes in mRNA levels, reflecting alterations in gene expression and/or mRNA stability. Higher expression levels of 17 genes were observed in the younger group, whereas increased expression of 7 genes was noted in the older group. Validation of the microarray findings was performed at the protein level using the Northern blot technique. Among the genes that were dominant in the retinas of the older participants was interleukin-1 (IL-1), a mediator of inflammation [42]. Elevated levels of IL-1 indicated that the aging process is associated with an inflammatory response. Additionally, somatostatin was found to have increased expression in the younger individuals, suggesting that this neuropeptide likely plays a crucial role in regulating retinal function [42]. These results were not confirmed in our study, possibly due to different research assumptions. Moreover, the reason for different results may be the type and origin of the test material. Yoshida et al. [42] examined material from deceased persons, which did not show phenotypic changes, and the donors were not treated for eye diseases. Furthermore, disparities in results may arise from variations in the methods used for result validation.

AMD is characterized by the formation and accumulation of deposits known as drusen. The presence of these deposits triggers inflammation, leading to an influx of inflammatory cells into the retinal pigment epithelial cells [43]. This in turn results in increased secretion of various growth factors, most notably VEGF [9,43]. VEGF is one of the most important activators of the neovascular form of AMD, making it a primary target for therapeutic intervention. Ranibizumab, a humanized monoclonal antibody fragment, effectively inhibits overactive VEGF-A molecules and is commonly used to treat this disease [44].

Sarraf et al. [45] studied the visual and anatomical outcomes in patients with neovascular AMD who were treated with ranibizumab over a 24-month period. They observed improvements in all patients, noting that higher concentrations of the drug yielded better anatomical outcomes [45]. In our research, we have also observed clinical improvements in patients, corroborating these earlier findings. despite using the same doses of the drug, contrary to the studies conducted by Sarraf et al. [45]. However, our study primarily focuses on the molecular aspects of the condition, and not on anatomical aspects as in the case of the above-mentioned study.

Another therapeutic approach aimed at inhibiting excessive VEGF activity is gene therapy. This involves inserting a complementary anti-VEGF DNA construct into photoreceptors and retinal pigment epithelial cells using a viral vector [46]. This technique enhances the production of VEGF inhibitors, reducing the need for intravitreal injections of medications, which can be burdensome for some patients. Clinical trials of this gene therapy have shown promising results: four out of six patients did not require further doses of the recombinant virus within the first year, and an improvement in vision was observed compared to their pre-therapy condition [46].

In the progression of AMD, another hallmark symptom is the development of severe maculopathy in Bruch’s membrane, followed by the accumulation of extracellular material. This accumulation ultimately leads to the death of photoreceptors and retinal epithelial cells [10,47]. Consequently, metalloproteinases, which are involved in remodeling the extracellular matrix, and their tissue inhibitors play a crucial role in the disease progression. Imbalances between these elements are responsible for pathological changes [48]. Administration of ranibizumab to patients with neovascular AMD resulted in an increase in the expression of some MMPs and a decrease in the amount of their inhibitor, which probably resulted in the breakdown of accumulated extracellular material and, as a result, led to an improvement in their health condition.

An increase in TIMP3 levels has been observed alongside a simultaneous decrease in the levels of MMP2 and MMP9 in individuals with AMD [49]. Hussain et al. [11] reported enhanced functionality of Bruch’s membrane in vitro following the addition of MMP2 and MMP9. In a separate study, Macgregor et al. [50] aimed to characterize the distribution of TIMP3 in human vascular tissues. Utilizing immunohistochemical staining in samples from 79 patients ranging in age from 18 months to 101 years, they found that TIMP3 levels increased with age and were present in Bruch’s membrane [50]. These results align with a study by Kamei and Hollifield [16], who also reported an increase in TIMP3 levels in Bruch’s membrane in AMD patients. In our study, however, we observed downregulation of only *TIMP2*. It is worth noting that our study focused on AMD patients before and after the administration of three doses of ranibizumab, and not on non-drug-stimulated samples. However, it should be remembered that the presented studies used other detection methods, i.e., Western blot, immunohistochemical analysis, or tissue microarrays [16]. The differences in results may also be influenced by the fact that in the cited studies, the samples came from deceased donors. Moreover, it is worth taking into account the age of the study group, the range of which was wide for the presented research, unlike in our own research.

In turn, Ecker et al. [51] showed a relationship between MMP9 expression and the level of subretinal fluid in patients with AMD. Their study employed a reverse-phase protein microarray and utilized fragments of the vitreous body as the test material [51]. In our own research, we observed alterations in the mRNA levels of various MMPs in AMD patients both before and during treatment with ranibizumab. We identified increased expression levels of *MMP11*, *MMP14*, *MMP15*, and *MMP24-A*. However, no significant changes in *MMP9* expression were observed. This discrepancy could stem from differences in methodology and the type of biological material analyzed.

*ADAMT* genes play a role in regulating proteolytic modifications within the retinal extracellular matrix. In particular, the *ADAMTS5* gene is linked to the retinal pigment epithelium, suggesting its potential involvement in AMD pathogenesis. Our own research revealed an upregulation of *ADAMTS5* gene expression, confirming previous findings such as those reported by Zhao et al. [20].

Brevitt et al. [52] also examined the expression patterns of ADAMT family genes in various ocular cell types, highlighting their role in AMD development, although they did not investigate PBMCs specifically. In contrast with their own study, the researchers conducted an analysis of the ARPE-19 cell line not treated with the drug. They used methods such as RT-PCR and Northern blot for analysis. Our study revealed differences in the expression profiles of genes encoding matrix metalloproteinases and their tissue inhibitors, including the upregulation of the *ADAMTS5* gene in patients with neovascular AMD treated with ranibizumab.

Based on our study results, one possible direction of change could be the impact of increased *MMP15* expression on ADAMTS protease activity. A study by Abu El-Asrar et al. [31] demonstrated that ADAMTS proteases can exhibit both pro- and anti-angiogenic effects, depending on their activity levels and concentrations. Additionally, ADAMTS proteases, including the significant ADAMTS-1, are linked to inflammation. TNF-alpha and lipopolysaccharides induce ADAMTS-1 in endothelial cells [31]. Nevertheless, considering the potential positive and negative consequences of increased ADAMTS activity, further research into this group of enzymes is warranted.

It is also possible that the observed decrease in *TIMP2* levels affects the inhibition of MMP2, which could have both positive and negative implications. A study by Hussain et al. [11] highlighted the dual nature of MMP2 activity in ocular disease. On one hand, inhibiting MMP2 activity with TIMP2 might be linked to increased deposit accumulation, while on the other hand, a decrease in this activity could have an anti-angiogenic effect [11]. Nevertheless, this issue requires further investigation. However, the exploratory study conducted has certain limitations. A relatively small number of samples from a small demographic area were analyzed. Moreover, the study included only people over 60 years of age, and the analysis was carried out for samples taken up to three months after the last injection, which may limit conclusions about the long-term effects of the study drug. The analysis techniques used also impose specific barriers on the results, e.g., they limit the number of analyzed changes in gene expression by determining the FC value of the microarray. However, despite this, in our opinion, the study conducted provides an opportunity to deepen knowledge of the effects of ranibizumab in patients with neovascular AMD and may constitute a starting point for further research in this area.

A detailed examination of the genes whose expression changed in our study could represent a significant breakthrough in unraveling the precise mechanisms underlying neovascular AMD pathogenesis. Initially, our focus could shift towards investigating the long-term effects of ranibizumab to validate our current findings and monitor the drug’s extended impact on the body. Furthermore, in the long run, our study may serve as a foundational point for exploring other forms of AMD and even other ocular diseases.

Furthermore, our exploratory study could serve as a foundational platform for more in-depth investigations into the alterations in gene expression discussed. Such research could pave the way for the development of a universal biomarker that signals the presence of AMD. This advancement might enable early disease detection and, by assessing the expression levels, facilitate the selection of optimal ranibizumab dosage and treatment strategies.

## 5. Conclusions

The administration of ranibizumab in patients with CNV/AMD modifies the systemic expression profiles of matrix metalloproteinase genes and their tissue inhibitors, indicating their potential role in treatment response. These findings provide valuable insights and could serve as a foundation for further in-depth investigations into the pathogenesis of AMD and various forms of this condition.

Notably, a significant increase in the mRNA level of *MMP15* emerges as a representative molecular marker in patients with the neovascular form of AMD. This observation may become crucial to the development of personalized ranibizumab treatment therapies for individual patients.

The data presented may serve as a useful reference point for future investigations involving larger groups of patients undergoing anti-VEGF therapy with a focus on paired comparisons. Additionally, the exploratory study primarily focused on the short-term effects of ranibizumab, without considering potential long-term therapy outcomes.

## Figures and Tables

**Table 1:** Demographic and clinical characteristics of study participants.

Neovascular AMD Patients (*n*)	Total 29
Gender (F/M) (*n*)	15/14
Age (years; mean ± SD)	76.8 ± 6.1
Female	78.2 ± 6.5
Male	79.5 ± 6.2
AMD in family	
Yes	0
No	29
Tabacco smoking	
Ever	17
Never	12

AMD—age-macular degeneration; *n*—number; F/M—female/male; SD—standard deviation.

**Table 2:** Sequences of primers used for real-time RT-qPCR reaction.

Gene	Oligonucleotide Sequence	Amplimer Length (bp)	Tm (°C)
*MMP15*	Forward: 5′GAAACAACCTCTTCCTGGTGGCAGTGCA3′ Reverse: 5′CGTGCCTCGGGCAGCTTGAAGTTGTC3′	135	84.6
*TIMP2*	Forward: 5′CCCCAAGCAGGAGTTTCTCGACATCG3′ Reverse: 5′TGGACCAGTCGAAACCCTTGGAGGCT3′	100	80.4
*ACTB*	Forward: 5′TCACCCACACTGTGCCCATCTACGA3′ Reverse: 5′CAGCGGAACCGCTCATTGCCAATGG3′	295	86.2

bp—base pairs; Tm—melting temperature.

**Table 3:** Number of ID mRNAs of metalloproteinase-related genes and their tissue inhibitors differentiating the tested transcriptomes according to the *p*-value.

FC	Number of ID mRNAs					
	*p* all	*p* < 0.05	*p* < 0.02	*p* < 0.01	*p* < 0.005	*p* < 0.001
FC ≥ 1.0	110	16	10	6	4	1
FC ≥ 2.0	9	6	3	2	1	0

FC—Fold change; lines highlighted in grey—statistic value used in the study.

**Table 4:** List of the transcripts associated with MMPs and TIMPs which showed a more than 2-fold statistically significant change in expression between the group before and 24 h after the third dose of the ranibizumab.

Probe ID	Gene Symbol	Gene Name	FC before vs. after	Change Direction
202828_s_at	*MMP14*	Matrix Metallopeptidase 14	2.20	↑
203167_at	*TIMP2*	TIMP Metallopeptidase Inhibitor 2	−2.68	↓
203365_s_at	*MMP15*	Matrix Metallopeptidase 15	2.41	↑
203876_s_at	*MMP11*	Matrix Metallopeptidase 11	2.19	↑
220705_s_at	*ADAMTS7*	ADAM Metallopeptidase With Thrombospondin Type 1 Motif 7	2.55	↑
221953_s_at	*MMP24A*	Matrix Metallopeptidase 24A	2.04	↑

FC—fold change (FC > 2.0); ↑—higher gene expression; ↓—lower gene expression; ID—the probe set identifier; before—the group before the administration of ranibizumab; after—the group after three injections of ranibizumab; lines highlighted in gray—differential transcripts whose expression was validated by RT-qPCR.

## Data Availability

Available on request and with regulations.
